# Supplementary material for: A hypothalamic circuit for circadian regulation of corticosterone secretion
Source: Nat Commun. 2026 Apr 7;17:4940. doi: 10.1038/s41467-026-71482-0 (PMC13233844; doi:10.1038/s41467-026-71482-0)
Supplement: Supplementary file 1 — Supplementary Information [file 41467_2026_71482_MOESM1_ESM.pdf]

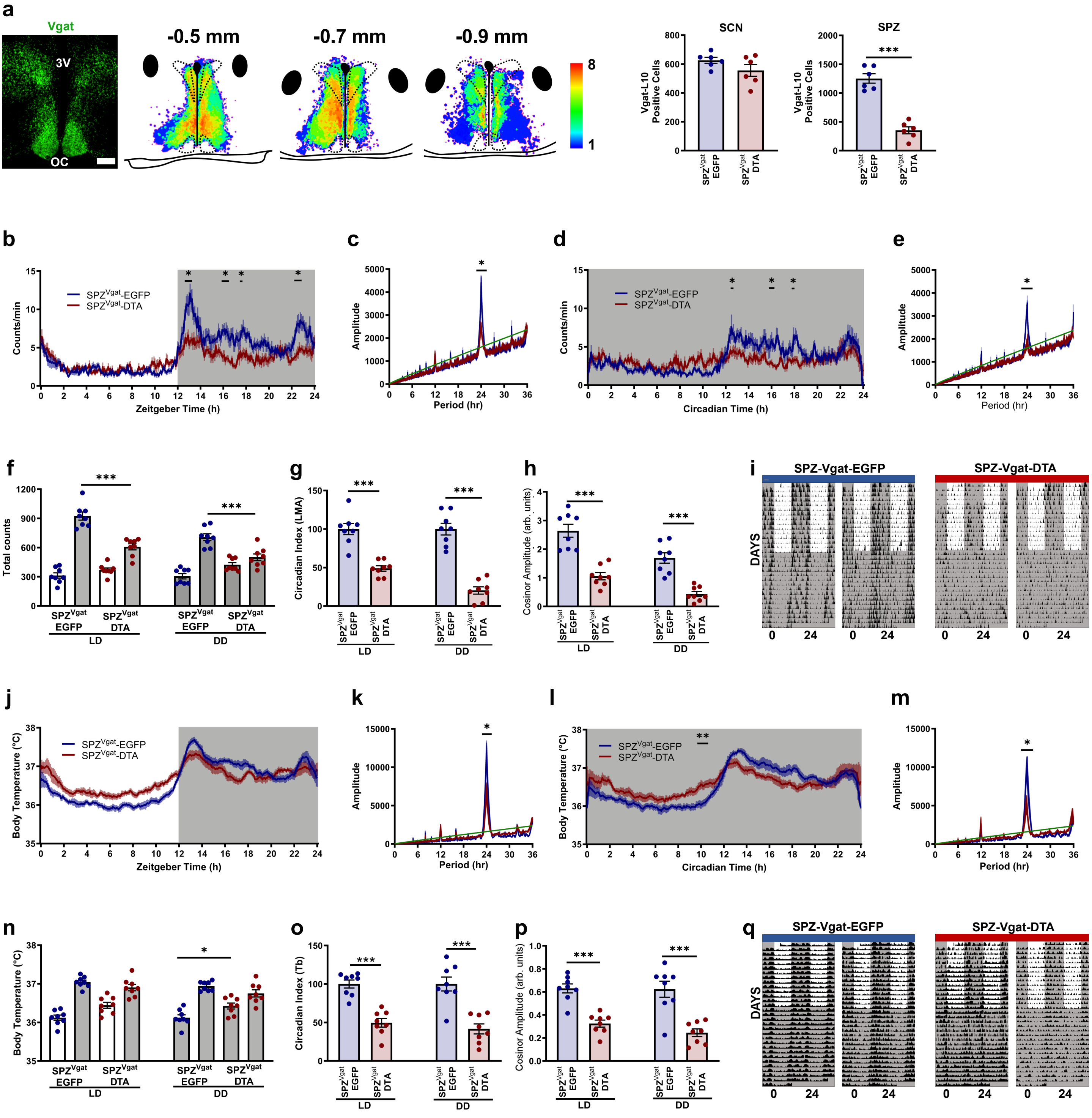

Supplementary Figure 1. SPZVgat neuron ablation flattens the circadian rhythm of LMA and reduces the amplitude of the rhythm of Tb. (a) Representative micrograph of GABAergic neurons (native signal in green) from a Vgat-ires-Cre::L10-EGFP control mouse (left panel); density plots of the distribution of injections of AAV-mCherry-DIO-DTA in the SPZ of the Vgat-ires-cre mice (n=8; middle panels); and counts of remaining L10-EGFP positive neurons in the SCN and SPZ (right panels, Unpaired t-test, Two-tailed: t=8.850, df=10, ***p<0.0001, n=6). (b) Daily LMA was reduced during the dark period in LD after SPZVgat ablation (Repeated Measures [RM] Two-way ANOVA; Šídák's multiple comparisons test. SPZVgat-EGFP vs SPZVgat-DTA: *p<0.05, ***p<0.001, n=8), and (c) the periodogram showed reduced amplitude (Two-way ANOVA; Šídák's multiple comparisons test. SPZVgat-EGFP vs SPZVgat-DTA: *p<0.05, n=8). (d) SPZVgat neuron ablation further reduced the circadian rhythm of LMA in DD (RM Two-way ANOVA; Šídák's multiple comparisons test. SPZVgat-EGFP vs SPZVgat-DTA: *p<0.05, n=8), (e) causing a dramatical reduction on the amplitude of the periodogram (Two-way ANOVA; Šídák's multiple comparisons test. SPZVgat-EGFP vs SPZVgat-DTA: *p<0.05, n=8). (f) Bar graphs showing total LMA counts in the light and dark periods from the SPZVgat-EGFP and SPZVgat-DTA mice. LMA was significantly reduced during the dark (Two-way ANOVA; Tukey's multiple comparisons: SPZVgat-EGFP dark vs SPZVgat-DTA dark = ***p<0.001, n=8) or subjective dark periods (Two-way ANOVA; Tukey's multiple comparisons: SPZVgat-EGFP subjective dark vs SPZVgat-DTA subjective dark = ***p<0.001, n=8). (g) The reduction of LMA during the dark or subjective dark period in SPZVgat-ablated mice reduced circadian index (CI) by 51.1 ±3.5% in LD (Unpaired t-test, Two-tailed: t=6.381, df=14, ***p<0.001, n=8) and by 79.7 ±4.8% in DD (Unpaired t-test, Two-tailed: t=8.901, df=14, ***p<0.001), (h) while the cosinor amplitude was also reduced in LD (Unpaired t-test, Two-tailed: t=6.142, df=14, ***p<0.001, n=8) and DD (Unpaired t-test, Two-tailed: t=6.190, df=14, ***p<0.001, n=8) (i) Representative LMA actograms, showing LD and DD recordings from SPZVgat-EGFP (blue) and SPZVgat-DTA (red) mice. (j-q) SPZVgat-ablated mice had a small increase in Tb during the light period and a small decrease during the dark period in both LD and DD, causing a significant change in the amplitude of the periodograms (LD: Two-way ANOVA; Šídák's multiple comparisons test. SPZVgat-EGFP vs SPZVgat-DTA: *p<0.05; DD: Two-way ANOVA; Šídák's multiple comparisons test. SPZVgat-EGFP vs SPZVgat-DTA: *p<0.05, n=8). However, (r) the CI was reduced by 50.4 ±5.6% in LD (Unpaired t-test, Two-tailed: t=6.551, df=14, ***p<0.00, n=81) and by 58.3 ±6.5% in DD (Unpaired t-test: t=5.228, df=14, ***p<0.001), and (s) the cosinor amplitude of Tb was also reduced both in LD (Unpaired t-test, Two-tailed: t=6.199, df=14, ***p<0.001, n=8) and DD (Unpaired t-test, Two-tailed: t=4.885, df=14, ***p<0.001, n=8). (t) Representative Tb actograms, red and blue lines as in panel i. LD, Light:Dark photoperiod; DD, Constant darkness; OC, Optic Chiasma; 3V, Third Ventricle. Reference scale bar: 200μm. Data are presented as mean and ± SEM. Gray shading indicates dark periods.

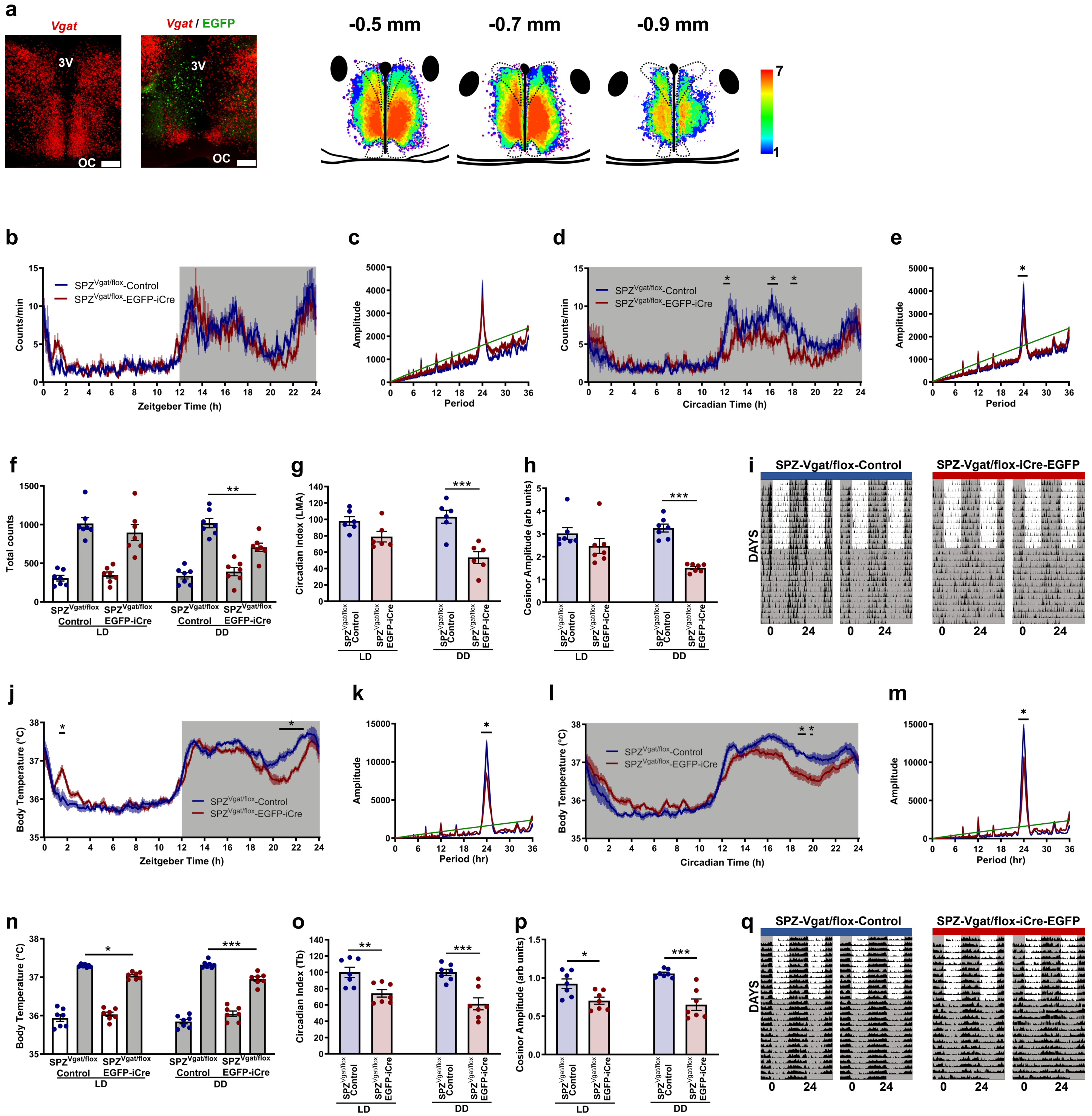

Supplementary Figure 2. Vgat gene deletion from SPZ neurons only partially reduces LMA and Tb during the dark period. (a) Representative micrograph of Vgat mRNA expression (in red) from a control mouse (left panel) and an iCre-EGFP injected mouse (in green; right panel). Notice the elimination of Vgat mRNA expression in the area where iCre-EGFP was expressed, whereas the Vgat expression in the SCN was preserved. The EGFP signal was enhanced with immunofluorescence for EGFP. Density plots of the distribution of injections of AAV-EGFP-iCre in the SPZ of the Vgat loxP/loxP mice (n=7; right panels). (b) Neither daily total LMA nor (c) the LMA periodogram was affected by the Vgat deletion in the SPZ when animals were in LD, but (d) LMA was reduced during the subjective dark period in DD (RM Two-way ANOVA; Šídák's multiple comparisons test. SPZVgat/flox-Control vs SPZVgat/flox-EGFP-iCre: *p<0.05, n=7), (e) with a reduction of the amplitude in the periodogram (Two-way ANOVA; Šídák's multiple comparisons test. SPZVgat/flox-Control vs SPZVgat/flox-EGFP-iCre: *p<0.05, n=7). (f) Total LMA counts in the light and dark periods from the SPZVgat/flox-Control and SPZVgat/flox-EGFP-iCre mice. Vgat ablation from SPZ neurons reduced the LMA during the subjective dark period under DD (Two-way ANOVA; Tukey's multiple comparisons: SPZVgat/flox-Control subjective dark vs SPZVgat/flox- EGFP-iCre subjective dark = **p=0.004, n=7), (g) resulting in a reduced circadian index by 39.7 ±4.8% in DD (Unpaired t-test, Two-tailed: t=4.674, df=10, ***p<0.001, n=7), (h) and a similar reduction the cosinor amplitude in DD (Unpaired t-test, Two-tailed: t=9.399, df=12, ***p<0.001, n=7) (i) Representative LMA actograms, showing LD and DD recordings from SPZVgat/flox-Control (blue) and SPZVgat/flox-EGFP-iCre (red). (j) The Vgat deletion from SPZ slightly reduced the Tb toward the end of the dark period in LD (RM Two-way ANOVA; Šídák's multiple comparisons test. SPZVgat/flox-Control vs SPZVgat/flox-EGFP-iCre: *p<0.05, n=7), (k) reducing the amplitude in the periodogram (Two-way ANOVA; Šídák's multiple comparisons test. SPZVgat/flox-Control vs SPZVgat/flox- EGFP-iCre: *p<0.05, n=7). (l) Similar reduction was observed during the subjective dark period in DD in the SPZVgat/flox-EGFP-iCre mice (RM Two-way ANOVA; Šídák's multiple comparisons test. SPZVgat/flox-Control vs SPZVgat/flox-EGFP-iCre: *p<0.05, n=7) (m) and in the periodogram (Two-way ANOVA; Šídák's multiple comparisons test. SPZVgat/flox-Control vs SPZVgat/flox-EGFP-iCre: *p<0.05, n=7). (n) The mean Tb was significantly reduced during the dark or subjective dark period in the SPZVgat/flox-EGFP-iCre mice either in LD (Two-way ANOVA; Tukey's multiple comparisons: SPZVgat/flox-Control vs SPZVgat/flox-EGFP-iCre dark = *p=0.021, n=7) or DD, (Two-way ANOVA; Tukey's multiple comparisons: SPZVgat/flox-Control subjective dark vs SPZVgat/flox-EGFP-iCre subjective dark = ***p<0.001, n=7). (o) The CI of Tb was reduced by 25.7 ±4.6% in LD (Unpaired t-test, Two-tailed: t=3.300, df=12, ***p=0.006, n=7) and by 38.6 ±7.3% in DD (Unpaired t-test, Two-tailed: t=4.689, df=12, ***p<0.001, n=7), (p) similar to the cosinor amplitude in LD (Unpaired t-test, Two-tailed: t=2.864, df=12, **p=0.014, n=7) and DD (Unpaired t-test, Two-tailed: t=5.318, df=12, ***p<0.001, n=7). (q) Representative Tb actograms, red and blue lines as in panel i. Data are presented as mean and ± SEM. Gray shading indicates dark periods.

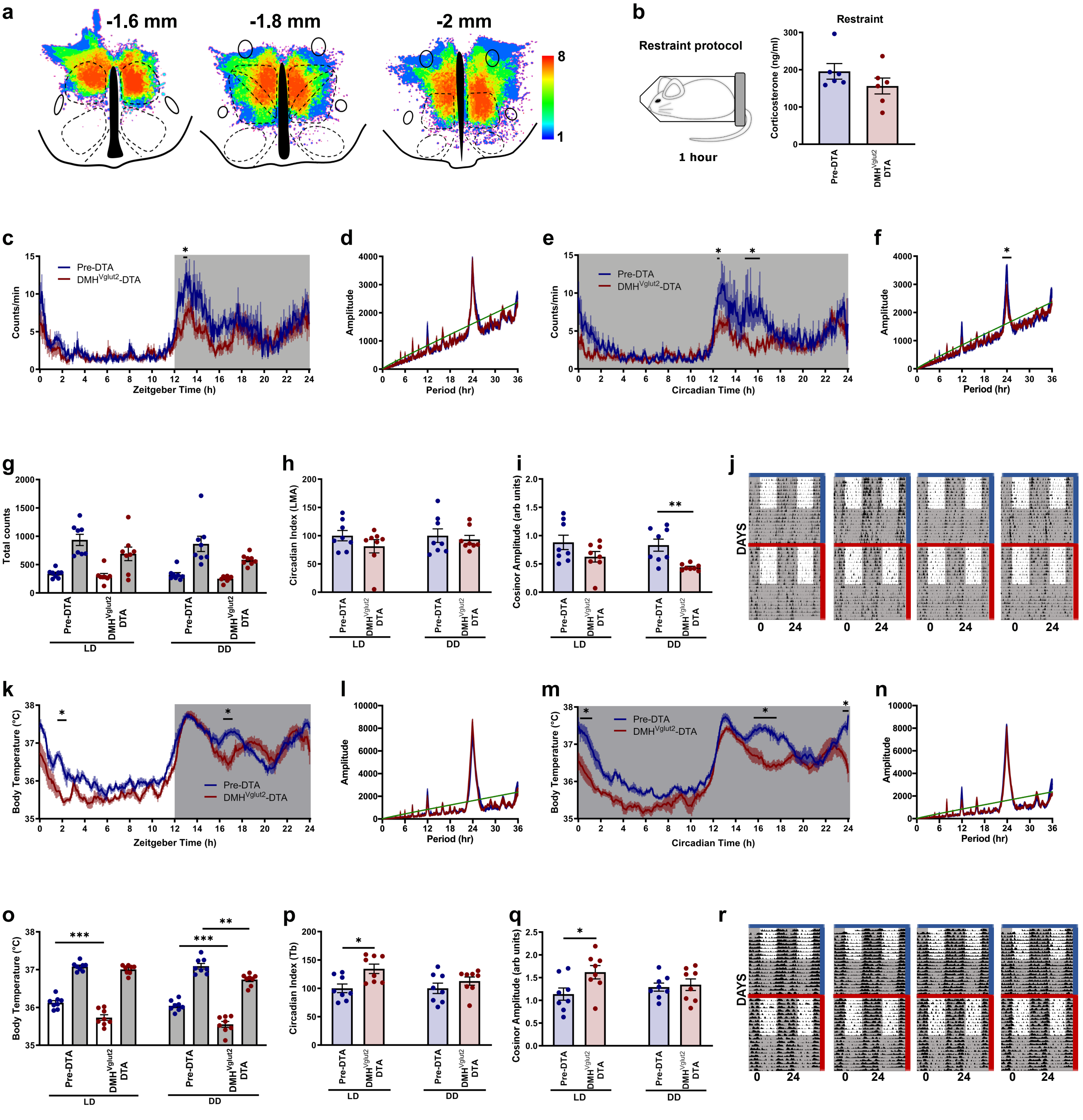

Supplementary Figure 3. DMHVglut2 neuron ablation reduces LMA during the subjective night and decreases Tb across the day. (a) Density plots of the distribution of injections of AAV-mCherry-DIO-DTA in the DMH of the Vglut2-ires-cre mice (n=8). (b) No statistically change was detected in the Cort levels in the restraint stress protocol. (c) Daily LMA was reduced during the dark period in LD after DMHVglut2 neuron ablation (RM Two-way ANOVA; Šídák's multiple comparisons test. Pre-DTA vs DMHVglut2-DTA: *p<0.05, n=8), but (d) the periodogram showed no change. (e) DMHVglut2 neuron ablation reduced LMA during the subjective dark in DD (RM Two-way ANOVA; Šídák's multiple comparisons test. Pre-DTA vs DMHVglut2-DTA: *p<0.05, n=8), (f) reducing the amplitude of the periodogram (Two-way ANOVA; Šídák's multiple comparisons test. Pre-DTA vs DMHVglut2-DTA: *p<0.05, n=8). (g) Bar graphs showing total LMA counts in the light and dark periods before and after ablation of DMHVglut2 neurons, during LD (left) and presumptive light and dark periods in DD (right). (h) The reduction of LMA during the dark period after DTA was sufficiently small that the circadian index was not significantly different. (i) However, the amplitude of the circadian rhythm of LMA as measured by cosinor analysis was reduced in DD after ablation (Paired t-test, Two-tailed: t=3.611, df=14, **p=0.002, n=8). (j) Representative LMA actograms, showing LD and DD recordings before (blue) and after (red) DTA. (k) Tb was reduced across the light phase and in the middle of the dark phase after DMHVglut2 neuron ablation in LD (Repeated Measures [RM] Two-way ANOVA; Šídák's multiple comparisons test. Pre-DTA vs DMHVglut2-DTA: *p<0.05, n=8), while (l) the periodogram showed no change. (m) The reduction in the daily Tb was larger in both the presumptive light and dark periods in DD after DMHVglut2 neuron ablation (RM Two-way ANOVA; Šídák's multiple comparisons test. Pre-DTA vs DMHVglut2-DTA: *p<0.05, n=8), (n) with no change in the periodogram. (o) The mean Tb was significantly reduced by DMHVglut2 ablation for the light but not the dark period in LD, and for both in DD (Two-way ANOVA; Tukey's multiple comparisons. ** p<0.01, ***p<0.001, n=8). However, (p) the CI and (q) the cosinor amplitude of Tb was increased after ablation (by 46.65 ±12.8%, n=8) only during LD (Paired t-test, Two-tailed, for CI: t=3.075, df=14, df=14, *p=0.008; for cosinor amplitude: t=2.415, df=14, *p=0.03, n=8). (r) Representative Tb actograms, red and blue lines as in panel j. LD, Light:Dark photoperiod; DD, Constant darkness. Data are presented as mean and ± SEM. Gray shading indicates dark periods.

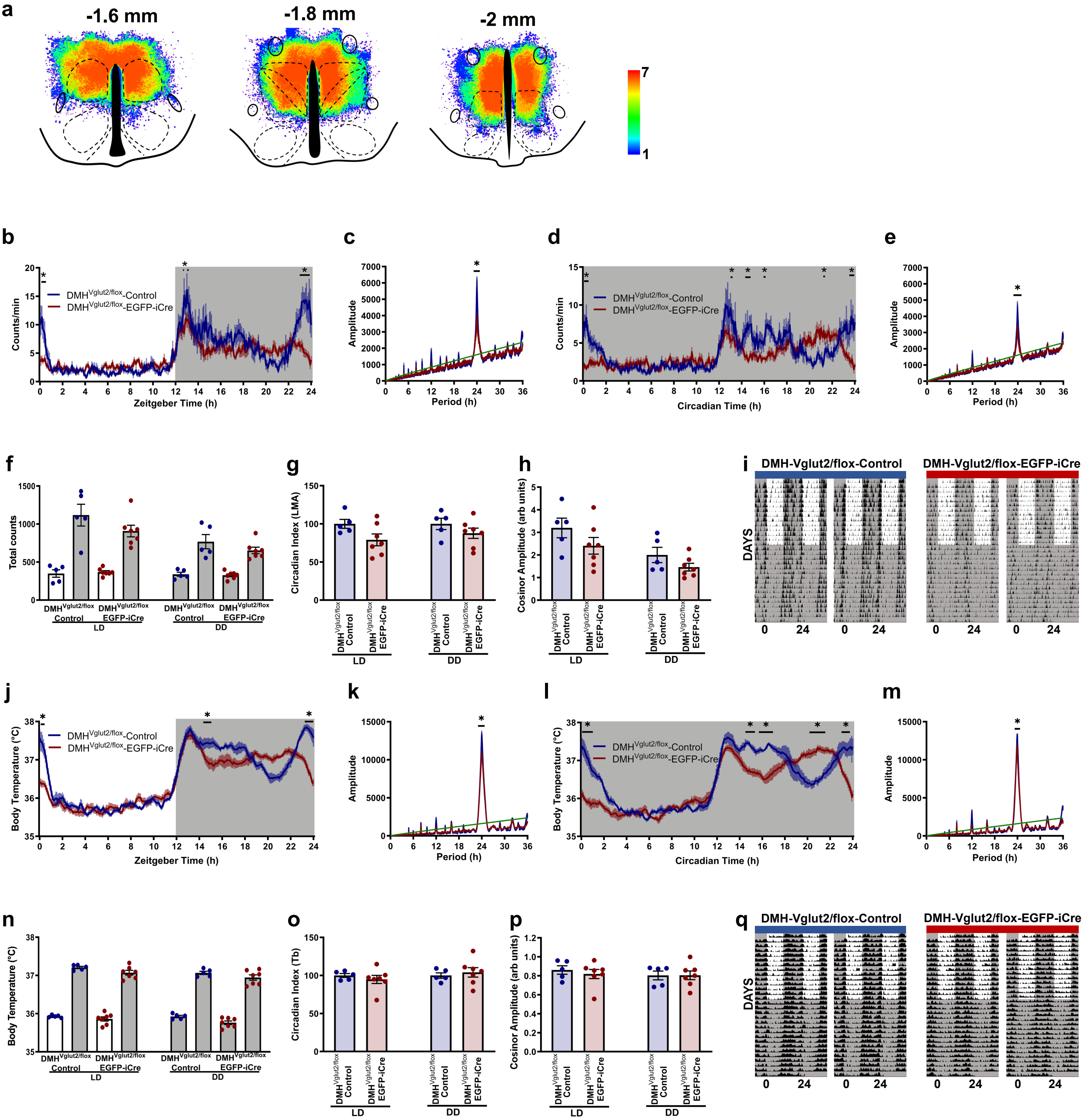

Supplementary Figure 4. Vglut2 gene deletion from DMH neurons reduces the peak of LMA and Tb during the transition from the dark to the light period. (a) Density plots of the injections of AAV-EGFP-iCre in the DMH of Vglut2loxP/loxP mice (n=7). (b) Daily distribution of LMA in LD (RM Two-way ANOVA; Šídák's multiple comparisons test. DMHVglut2/flox-Control vs DMHVglut2/flox-EGFP-iCre: *p<0.05, n=8). Vglut2 gene deletion from DMH neurons reduced LMA during the transition from the dark to the light period, and to a lesser extend in the early dark period, (c) reducing the amplitude of the periodogram peak at 24h (Two-way ANOVA; Šídák's multiple comparisons test. DMHVglut2/flox-Control vs DMHVglut2/flox-EGFP-iCre: *p<0.05, n=8). (d) In DD, the reduction in LMA during the transitions between the presumptive light and dark periods was similar to LD (RM Two-way ANOVA; Šídák's multiple comparisons test. DMHVglut2/flox-Control vs DMHVglut2/flox-EGFP-iCre: *p<0.05, n=8), (e) with similar reduction in the amplitude of the periodogram (Two-way ANOVA; Šídák's multiple comparisons test. DMHVglut2/flox-Control vs DMHVglut2/flox-EGFP-iCre: *p<0.05, n=8). (f) However, for the entire light and dark periods in LD and presumptive light and dark periods in DD, the small changes in LMA did not reach statistical significance for total LMA counts, (g) CI or (h) cosinor amplitude. (i) Representative LMA actograms from the Control and DMH Vglut2 gene-deleted mice. (j) Tb was also reduced in LD during the transition from the dark to the light phase and to a lesser extent in the early dark phase in DMH Vglut2 gene-deleted mice (RM Two-way ANOVA; Šídák's multiple comparisons test. DMHVglut2/flox-Control vs DMHVglut2/flox-EGFP-iCre: *p<0.05, n=8), (k) reducing the amplitude in the periodogram around the 24 period (Two-way ANOVA; Šídák's multiple comparisons test. DMHVglut2/flox-Control vs DMHVglut2/flox-EGFP-iCre: *p<0.05, n=8). (l) A similar pattern in Tb was seen during DD (RM Two-way ANOVA; Šídák's multiple comparisons test. DMHVglut2/flox-Control vs DMHVglut2/flox-EGFP-iCre: *p<0.05, n=8), (m) with similar reduction in the amplitude of the periodogram (Two-way ANOVA; Šídák's multiple comparisons test. DMHVglut2/flox-Control vs DMHVglut2/flox-EGFP-iCre: *p<0.05, n=8). (n) However, as for LMA, these changes were not large enough to produce a statistically significant difference in mean Tb, (o) CI or (p) cosinor amplitude of the circadian rhythm of Tb. (q) Tb actograms from the DMH Vglut2 gene-deleted mice included in this study. LD, Light:Dark photoperiod; DD, Constant darkness. Data are presented as mean and ± SEM. Gray shading indicates dark periods.

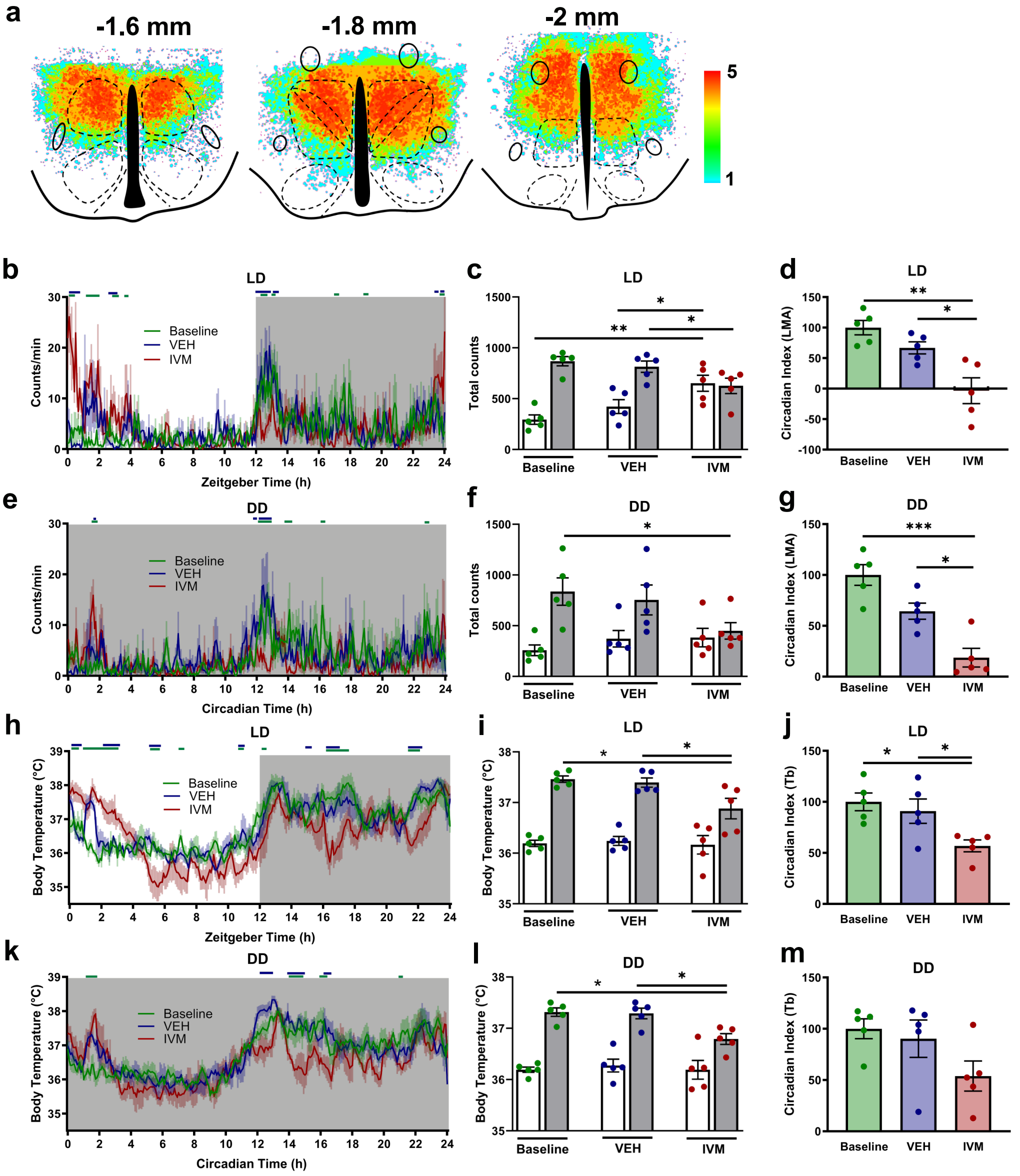

Supplementary Figure 5. Chemogenetic inhibition of the DMHVglut2 neurons decreases the circadian index of LMA and reduces Tb. (a) Density plots of the distribution of injections of AAV-DIO-hGlyR-mCherry in the DMH of the Vglut2-ires-Cre mice (n=5). (b-d) Chemo-inhibitions with IVM in LD induced higher LMA at the transition from the dark to the light phase and reduced the peak during the first hours of the dark phase (b: RM Two-way ANOVA; Tukey's multiple comparisons test p<0.05. Line above the 24h graphs represent significative differences in Baseline vs IVM in green, and VEH vs IVM in blue; c: Two-way ANOVA; Tukey's multiple comparisons test. light Baseline vs light IVM: **p=0.001, light VEH vs light IVM: *p=0.042, dark Baseline vs dark IVM: *p=0.03, n=5), reducing the CI (d: One-way ANOVA; Tukey's multiple comparisons test. Baseline vs IVM: **p=0.001, VEH vs IVM: *p=0.016, n=5). (e-g) In DD, the daily increase in LMA at the beginning of the dark period was reduced (e: RM Two-way ANOVA; Tukey's multiple comparisons test p<0.05. Lines above same as in B. f: Two-way ANOVA; Tukey's multiple comparisons test. dark Baseline vs dark IVM: *p=0.036, n=5), reducing also the LMA CI (g: One-way ANOVA; Tukey's multiple comparisons test. Baseline vs IVM: ***p<0.001, VEH vs IVM: *p=0.01, n=5). (h-m) Tb during the dark and presumptive dark period was reduced between 24-48 hr after IVM (h and k: RM Two-way ANOVA; Tukey's multiple comparisons test p<0.05. Line above same as in B. I: Two-way ANOVA; Tukey's multiple comparisons test. dark Baseline vs dark IVM: *p=0.01, dark VEH vs dark IVM: *p=0.024. l: Two-way ANOVA; Tukey's multiple comparisons test. dark Baseline vs dark IVM: *p=0.01, dark VEH vs dark IVM: *p=0.014, n=5) resulting in a roughly 50% decrease in the CI of Tb during this same time period in LD and DD, although this only reached statistical significance in LD (One-way ANOVA; Tukey's multiple comparisons test. Baseline vs IVM: *p=0.015, VEH vs IVM: *p=0.024, n=5). Data are presented as mean and ± SEM. Gray shading indicates dark periods.

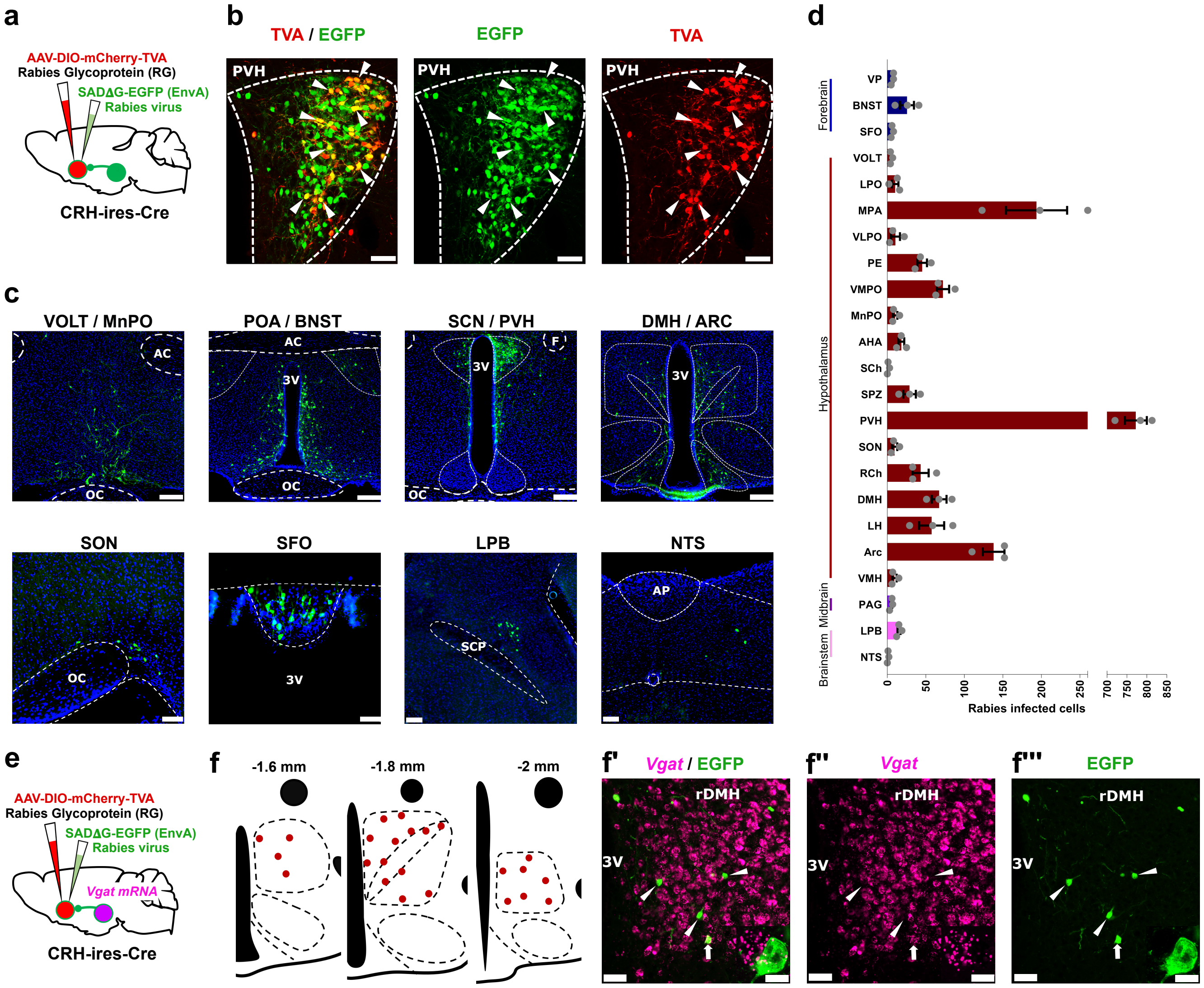

Supplementary Figure 6. Presumed monosynaptic inputs to the PVHCRH neurons based on conditional rabies virus tracing. (a) Schematic of the rabies infection of PVHCRH neurons to map their inputs. (b) Expression of both TVA (in red) and rabies EnvA (in green) marks doubly-transfected PVHCRH neurons (yellow) as "starter cells" to which neurons labeled only with green are presumed to project. (c) Representative micrographs of areas with retrogradely labeled neurons. (d) Total counts of the EnvA-rabies transfected neurons through the brain (n=3, mean and ± SD). The hypothalamus represents the most important source of inputs to the PVHCRH neurons. (e) Schematic of the EnvA-rabies experiment to map the monosynaptic input from the DMHVgat neurons to PVHCRH neurons. (f) Mapping of the rabies-Vgat co-labeling distribution in the DMH at different rostro-caudal levels (left panel), and representative images showing Vgat mRNA expression (in magenta, f' and f'') and rabies expression (in green; f' and f''') within the DMH (right panels). Neurons without Vgat mRNA (presumably glutamatergic) are shown by arrowheads and a doubly labeled cell indicated by the arrow is shown in a magnified inset at the lower right of each panel. The green signal from the Rabies infected cells was enhanced with immunofluorescence for EGFP. Reference scale bar: in c = 200μm, in b and f'-f'''= 50μm, in f'-f''' insets = 10μm. 3V, third ventricle; AC, Anterior commissure; F, Fornix; OC, Optic Chiasm; SCP, Superior Cerebellar Peduncle; AHA, Anterior Hypothalamic Area; Arc, Arcuate Nucleus; BNST, Bed Nucleus of the Stria Terminalis; DMH, Dorsomedial Hypothalamus; LH, Lateral Hypothalamus; LPB, Lateral Parabrachial; LPO, Lateral Preoptic Area; MnPO, Median Preoptic Nucleus; MPA, Medial Preoptic Area; NTS, Nucleus of the Tractus Solitarius; PAG, Periaqueductal Gray Area; PE, Periventricular hypothalamic nucleus; POA, Preoptic Area; PVH, Paraventricular Hypothalamic nucleus ; RCh, Retrochiasmatic Nucleus; SCN, Suprachiasmatic Nucleus; SFO, Subfornical organ; SON, Supraoptic Nucleus; SPZ, Subparaventricular Zone; VLPO, Ventrolateral Preoptic Area; VMH, Ventromedial Hypothalamus; VMPO, Ventromedial Preoptic Area; VOLT, Vascular Organ of Lamina Terminalis; VP, Ventral Pallidum. Neuroanatomical regions and names were based on the Paxinos & Franklin Atlas.

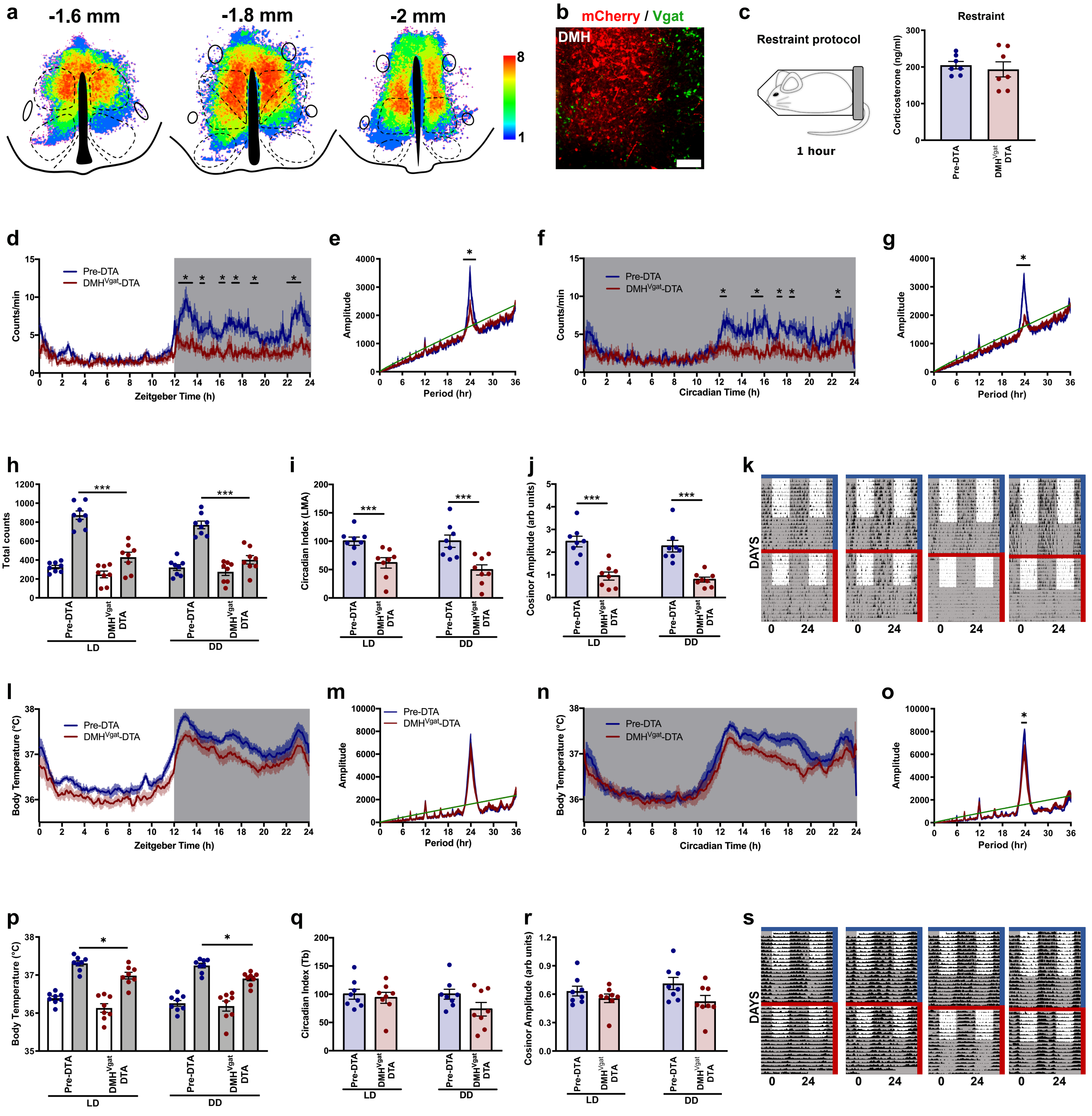

Supplementary Figure 7. Ablation of DMHVgat neurons dramatically reduces the total amount and circadian rhythm of LMA, but only reduces the daily level of Tb with little effect on its circadian rhythm. (a) Density plots of the distribution of injections of AAV-mCherry-DIO-DTA in the DMH of Vgat-ires-cre mice (n=8). (b) Magnification of the representative micrograph showed in Fig 5b, showing few if any remaining Vgat-expressing neurons (green, native signal) within the area of the injection site (red, native signal). (c) The Cort levels were similar in the restraint stress protocol. (d-j) LMA was reduced during the dark phase after DMHVgat neuron ablation in both LD (RM Two-way ANOVA; Šídák's multiple comparisons test. Pre-DTA vs DMHVgat-DTA: *p<0.05, n=8) and DD (RM Two-way ANOVA; Šídák's multiple comparisons test. Pre-DTA vs DMHVgat-DTA: *p<0.05, n=8), resulting in a reduced circadian index by 38.23 ±9.2% in LD (Paired t-test, Two-tailed: t=3.252, df=14, ***p=0.005, n=8) and by 50.61 ±12.9% in DD (Paired t-test: t=3.605, df=14, ***p=0.003, n=8) and similar reductions in cosinor amplitude in LD (Paired t-test, Two-tailed: t=5.138, df=14, ***p<0.001, n=8) and DD (Paired t-test, Two-tailed: t=5.503, df=14, ***p<0.001, n=8), with a reduction in peak amplitude but no change in tau in the periodogram (LD and DD: Two-way ANOVA; Šídák's multiple comparisons test. Pre-DTA vs DMHVgat-DTA: *p<0.05, n=8). (k) Representative LMA actograms before and after DMHVgat neuron ablation (blue lines represents pre-ablation and red lines is post-ablation). (l) Tb was reduced by about 0.3o C in LD, but (m) there was no change in the amplitude or period of the rhythm in periodogram analysis. (n) In DD, only the Tb during the presumptive dark period was reduced, but (o) this reduced the amplitude of the mean periodogram peak at 24h (Two-way ANOVA; Šídák's multiple comparisons test. Pre-DTA vs DMHVgat-DTA: *p<0.05, n=8). (p) The reduction in mean Tb during the dark period is statistically significant in both LD and DD (LD: Two-way ANOVA; Tukey's multiple comparisons. Dark Pre-DTA vs DMHVgat-DTA: *p=0.032; DD: Dark Pre-DTA vs DMHVgat-DTA: *p=0.012, n=8), but (q-r) there is no significant difference in the CI or cosinor amplitude of Tb. (s) Representative Tb actograms before and after DMHVgat neuron ablation. Data are presented as mean and ± SEM. Gray shading indicates dark periods.

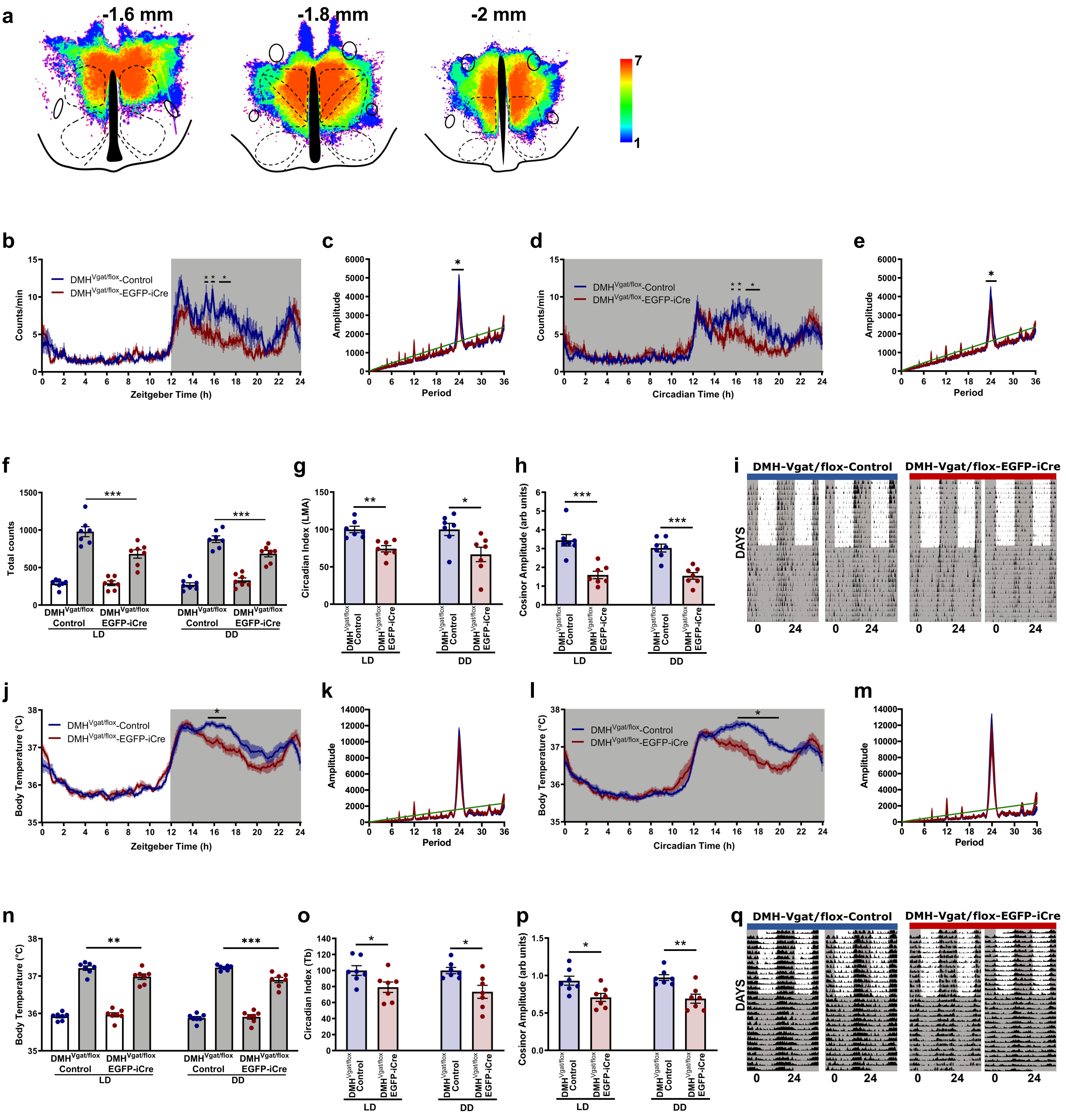

Supplementary Figure 8. Vgat gene deletion in DMH neurons reduces the elevation of LMA and Tb during the middle of the dark and presumptive dark periods and the amplitude of their circadian rhythms. (a) Density plots of the injections of AAV-EGFP-iCre in the DMH of VgatloxP/loxP mice (n=7). (b-e) Vgat gene deletion in the DMH caused lower LMA during the middle of the dark phase in LD and presumptive dark phase in DD (LD: RM Two-way ANOVA; Šídák's multiple comparisons test. DMHVgat/flox-Control vs DMHVgat/flox-EGFP-iCre: *p<0.05; DD: RM Two-way ANOVA; Šídák's multiple comparisons test. DMHVgat/flox-Control vs DMHVgat/flox-EGFP-iCre: *p<0.05, n=7) with a reduction in the amplitude of in the mean periodogram peak at 24h (LD: Two-way ANOVA; Šídák's multiple comparisons test. DMHVgat/flox-Control vs DMHVgat/flox-EGFP-iCre: *p<0.05; DD: Two-way ANOVA; Šídák's multiple comparisons test. DMHVgat/flox-Control vs DMHVgat/flox-EGFP-iCre: *p<0.05, n=7). (f) Deletion of the Vgat gene in the DMH reduced movement during the dark or subjective dark period (Two-way ANOVA; Tukey's multiple comparisons test. LD DMHVgat/flox-Control vs DMHVgat/flox-EGFP-iCre: ***p<0.001; DD Dark DMHVgat/flox-Control vs DMHVgat/flox-EGFP-iCre: ***p<0.001, n=7). (g) The CI of LMA rhythm was reduced by 25.73 ±6.01% in LD (Unpaired t-test, Two-tailed: t=4.275 df=12, **p=0.001, n=7) and 33.35 ± 12.63% in DD (Unpaired t-test, Two-tailed: t=2.642 df=12, *p=0.021, n=7) and (h) the cosinor amplitude of LMA was similarly reduced in LD (Unpaired t-test, Two-tailed: t=5.194, df=12, ***p<0.001, n=7) and DD (Unpaired t-test, Two-tailed: t=5.232, df=12, ***p<0.001, n=7). (i) Representative LMA actograms from AAV-DIO-EGFP and AAV-DIO-EGFP-iCre injected mice. (j-m) Tb is reduced during the middle of the dark phase in DMH Vgat gene-deleted mice in LD (RM Two-way ANOVA; Šídák's multiple comparisons test. DMHVgat/flox-Control vs DMHVgat/flox-EGFP-iCre: *p<0.05, n=7) and the presumptive dark phase in DD (RM Two-way ANOVA; Šídák's multiple comparisons test. DMHVgat/flox-Control vs DMHVgat/flox-EGFP-iCre: *p<0.05, n=7), with no change in the mean periodogram. (n) The reduction in mean Tb during the dark and presumptive dark phase in the DMH Vgat-gene deleted mice was statistically significant (Two-way ANOVA; Tukey's multiple comparisons test. LD Dark DMHVgat/flox-Control vs DMHVgat/flox-EGFP-iCre: **p=0.001; DD Dark DMHVgat/flox-Control vs DMHVgat/flox-EGFP-iCre: ***p<0.001, n=7). (o) As a result, the CI of Tb was also reduced in the DMH Vgat-gene deleted mice by 20.84 ±8.8% in LD (Unpaired t-test, Two-tailed: t=2.342 df=12, *p=0.037, n=7) and by 26.6 ±8.9% under constant dark (Unpaired t-test, Two-tailed: t=2.958 df=12, *p=0.012, n=7), and (p) the cosinor amplitude was lower in DMH Vgat gene-deleted mice in LD (Unpaired t-test, Two-tailed: t=2.482, df=12, *p= 0.028, n=7) and DD (Unpaired t-test, Two-tailed: t=3.902, df=12, **p=0.002, n=7). (q) Representative Tb actograms from AAV-DIO-EGFP and AAV-DIO-EGFP-iCre injected mice. Data are presented as mean and ± SEM. Gray shading indicates dark periods.

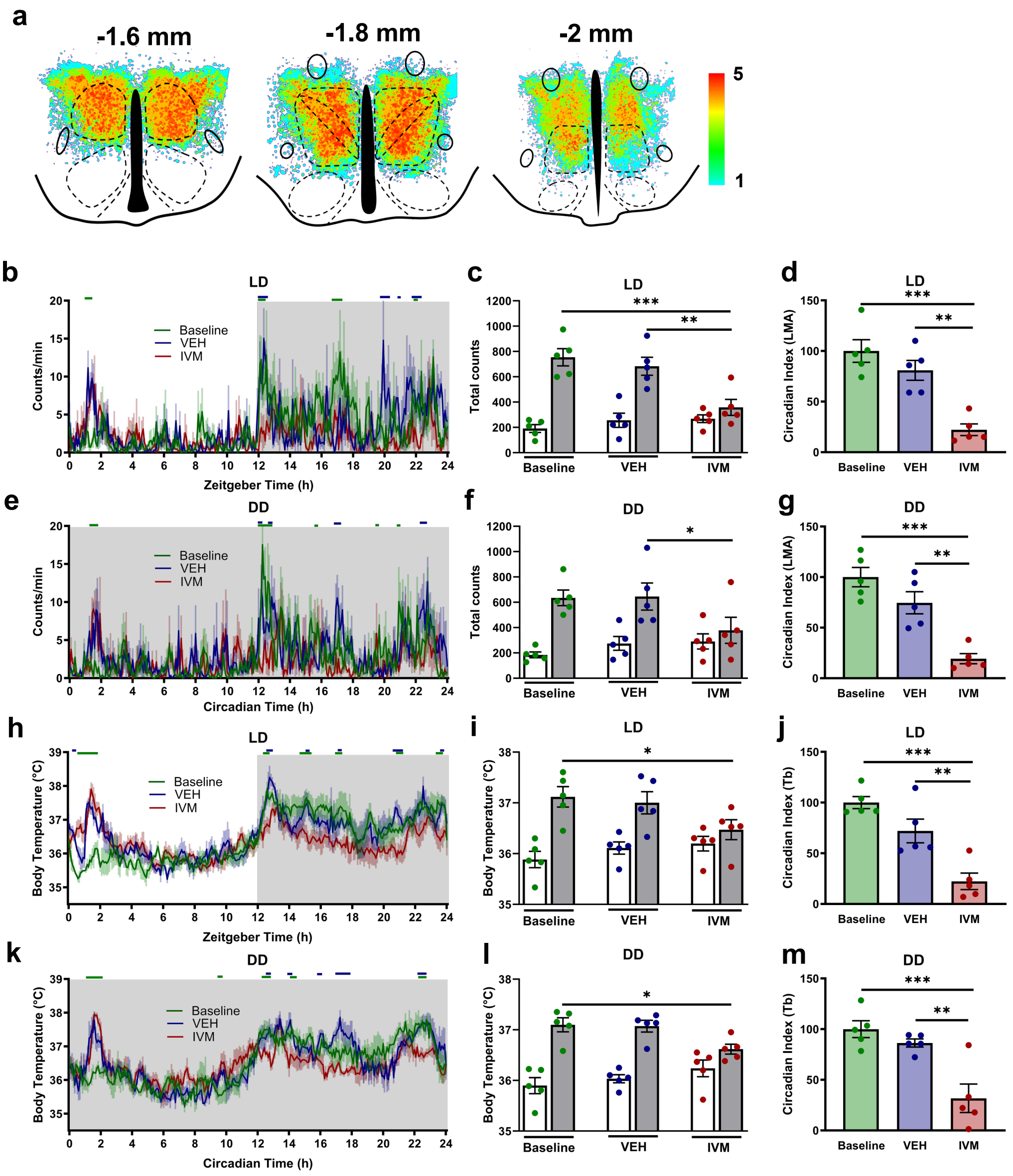

Supplementary Figure 9. Chemogenetic inhibition of DMHVgat neurons flattens the circadian rhythm of LMA and Tb. (a) Density plots of the AAV-DIO-hGlyR-mCherry injection sites in the DMH of Vgat-ires-Cre mice (n=5). (b-g) There was a reduction in the amount of LMA during the dark and presumptive dark periods between 24-48 hr after IVM injection (b and e: RM Two-way ANOVA; Tukey's multiple comparisons test p<0.05. Line above the 24h graphs represent significative differences in Baseline vs IVM in green, and VEH vs IVM in blue; c: Two-way ANOVA; Tukey's multiple comparisons test. dark Baseline vs dark IVM: ***p<0.001, dark VEH vs dark IVM: **p=0.001. F: Two-way ANOVA; Tukey's multiple comparisons test. dark VEH vs dark IVM: *p=0.04, n=5), resulting in a dramatic reduction in CI during the same time period (d: One-way ANOVA; Tukey's multiple comparisons test. Baseline vs IVM: ***p<0.001, VEH vs IVM: **p=0.001. g: One-way ANOVA; Tukey's multiple comparisons test. Baseline vs IVM: ***p<0.001, VEH vs IVM: **p=0.002, n=5). (h-m) Reductions in mean Tb after IVM administration were observed during the dark and subjective dark periods (h and k: RM Two-way ANOVA; Tukey's multiple comparisons test p<0.05. i: Two-way ANOVA; Tukey's multiple comparisons test. dark Baseline vs dark IVM: *p=0.041. l: Two-way ANOVA; Tukey's multiple comparisons test. dark Baseline vs dark IVM: *p=0.038, n=5), driving a reduction in CI in both LD and DD (j: One-way ANOVA; Tukey's multiple comparisons test. Baseline vs IVM: ***p<0.001, VEH vs IVM: **p=0.005. m: One-way ANOVA; Tukey's multiple comparisons test. Baseline vs IVM: ***p<0.001, VEH vs IVM: **p=0.004, n=5). Data are presented as mean and ± SEM. Gray shading indicates dark periods.

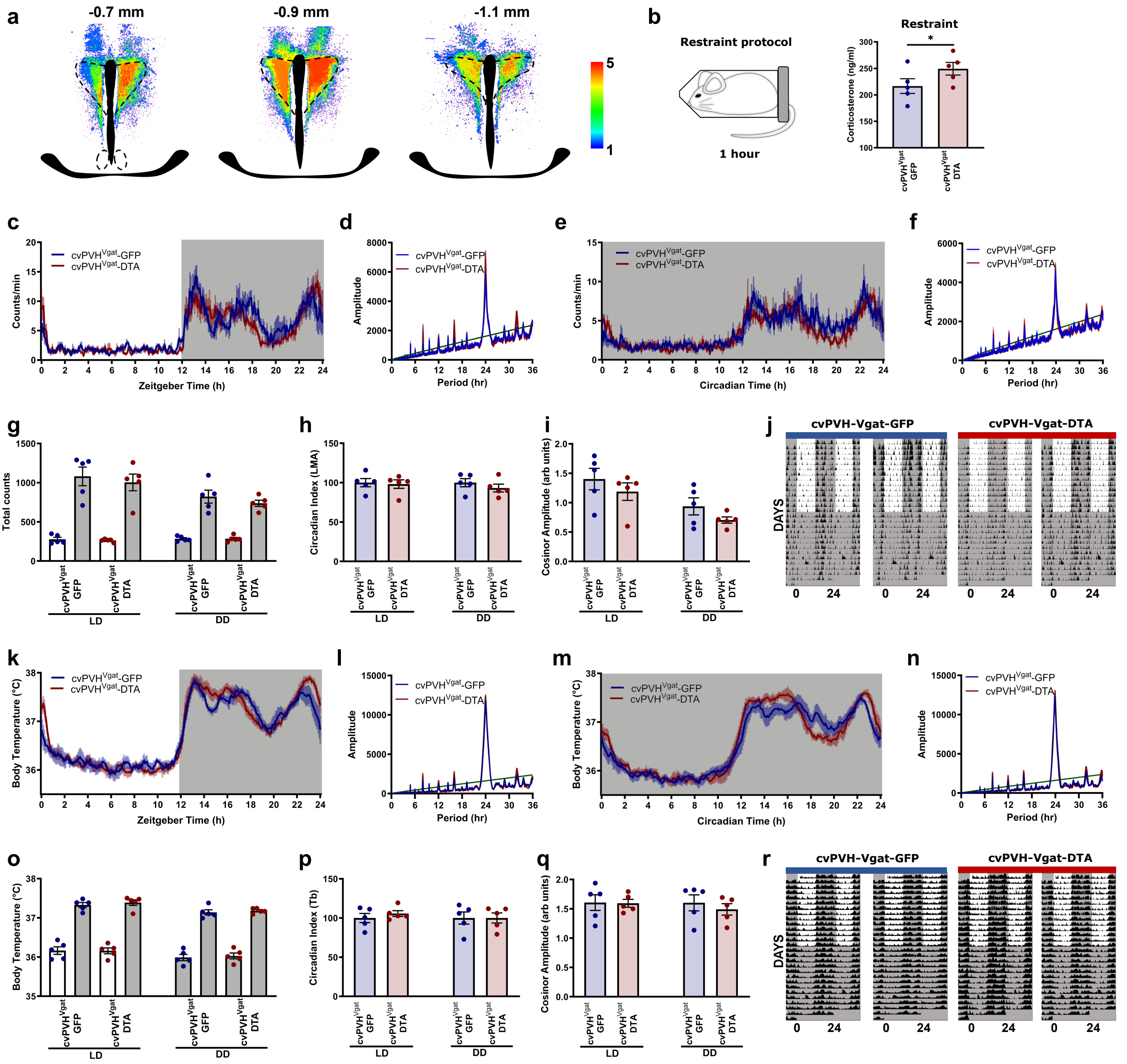

Supplementary Figure 10. cvPVHVgat neuron ablation increases the Cort response to restraint stress, but does not affect the circadian rhythm of LMA or Tb. (a) Density plots showing the distribution of injections of AAV-mCherry-DIO-DTA in the cvPVH of Vgat-ires-Cre mice (n=5). (b) After 1 hour of movement restraint, mice with cvPVHVgat ablation had higher levels of Cort (Unpaired t test: t=2.782, df=8, p= 0.047, n=5). (c) The 24h LMA counts and (d) mean periodogram under LD, as well as under DD (e, f) did not differ from control mice. (g-i) As a result, no differences were detected in the circadian rhythms of LMA after cvPVHVgat neuron ablation (j) Representative LMA actograms from both controls and mice with ablation of cvPVHVgat neurons. (k-n) There were no significant changes in mean Tb levels or their periodograms under either LD or DD. (o-q) Likewise, the circadian rhythm of Tb was undisturbed by the cvPVHVgat ablation. (r) Representative Tb actograms from Vgat-Cre animals with injections of AAV-DIO-EGFP and AAV-mCherrry-DIO-DTA the cvPVH. LD, Light:Dark photoperiod; DD, Constant darkness. Data are presented as mean and ± SEM. Gray shading indicates dark periods.

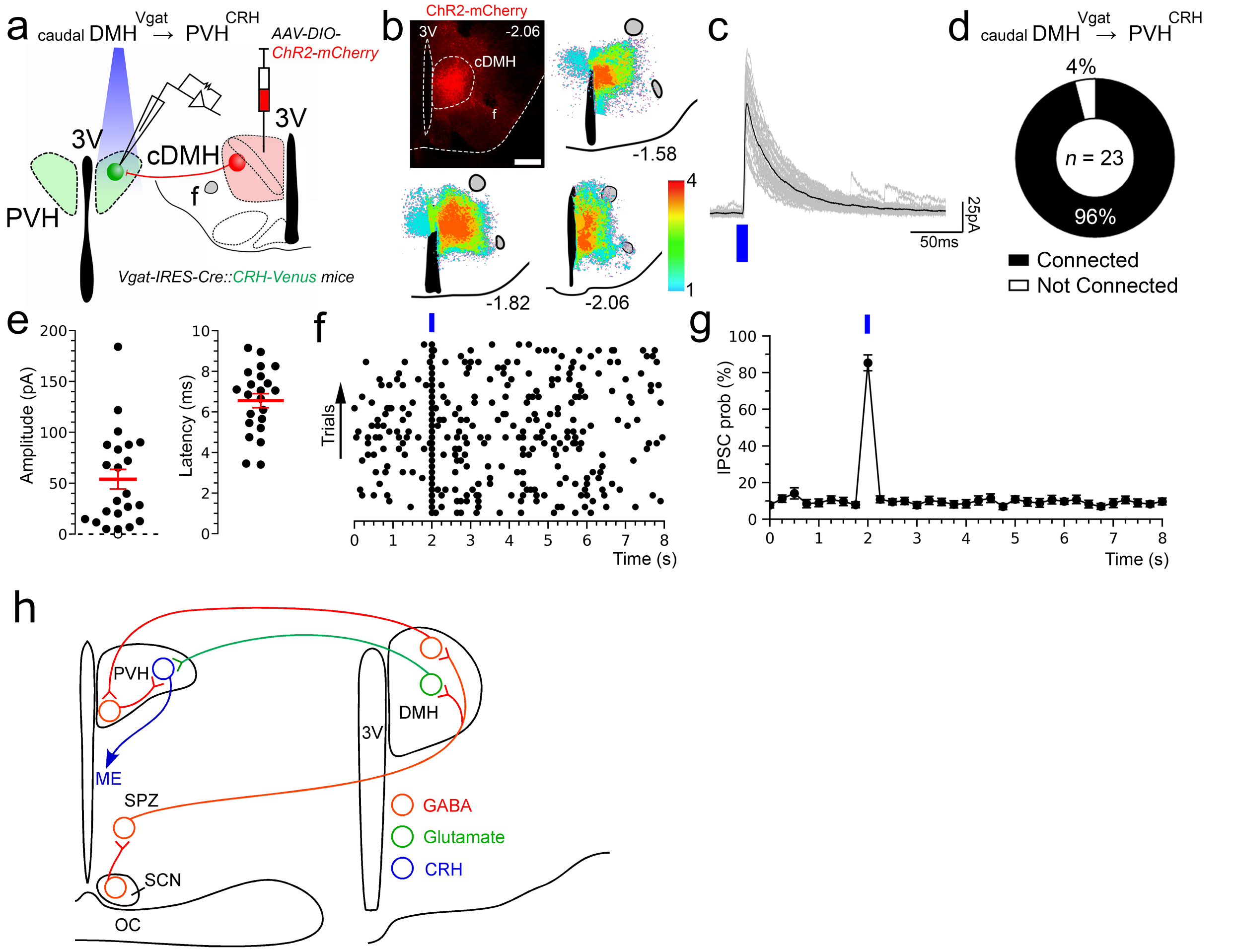

Supplementary Figure 11. In vitro optogenetic stimulation of the GABAergic input from the caudal DMH inhibits PVHCRH neurons. (a) A schematic of the experiment demonstrating connectivity between the caudal DMH (cDMH) Vgat neurons and ipsilateral PVHCRH neurons (cDMHVgat → PVHCRH; the DMH is shown on the opposite side of the brain to ease illustration). Vgat-ires-Cre::CRH-Venus mice were injected with AAV-DIO-ChR2-mCherry in the cDMH, and recordings were conducted in brain slices from Venus-labeled PVHCRH neurons while photostimulating the cDMHVgat input. (b) An example of ChR2-mCherry expression in the cDMH (top left, native signal) and density plots of the AAV-DIO-ChR2-mCherry injection sites (n = 4 mice; right and bottom). (c) Opto-evoked inhibitory post-synaptic currents (oIPSCs) recorded in the PVHCRH neurons. (d) Percentages of PVHCRH neurons responding (Connected) and not responding (Not Connected) to photostimulation of the cDMHVgat input (n= 23 PVHCRH recorded neurons from 4 mice). (e) Amplitude (left; filled markers, cells responding to photostimulation, n=22, open markers, cells not responding to photostimulation, n=1 neurons, from 4 mice; mean and ± SEM of responding neurons) and latency (right) of oIPSCs in PVHCRH neurons in response to photostimulation of the cDMHVgat input (mean and ± SEM; n=22 from 4 mice). (f) Raster plot of IPSCs in a representative PVHCRH neurons with photostimulation of the cDMHVgat → PVHCRH input (bin duration: 50ms). (g) IPSC probability in response to photostimulation of the cDMHVgat → PVHCRH input (black, n = 23). (h) A summary diagram to illustrate the network for circadian control of corticosteroid secretion. The circadian information from the SCN has an obligate relay in the SPZ, which projects to the DMH. Glutamatergic DMH neurons (green) directly activate CRH neurons (blue) in the PVH to cause the daily surge in CORT before the active phase. GABAergic neurons (red) in the DMH Innervate other GABAergic neurons in the caudoventral PVH, which in turn innervate the PVH CRH neurons. This circuit is necessary to disinhibit the CRH neurons during the daily surge in CORT levels. CRH, corticotrophin-releasing hormone; DMH, dorsomedial hypothalamic nucleus; ME, median eminence; OC, optic chiasm; PVH, paraventricular hypothalamic nucleus; SCN, suprachiasmatic nucleus; SPZ, subparaventricular zone. Reference scale bar: in (b) = 250 μm. f, fornix; 3V, third ventricle.
