# Supplementary material for: A hypothalamic circuit for circadian regulation of corticosterone secretion
Source: Nat Commun. 2026 Apr 7;17:4940. doi: 10.1038/s41467-026-71482-0 (PMC13233844; doi:10.1038/s41467-026-71482-0)
Supplement: Supplementary file 2 — Reporting Summary [file 41467_2026_71482_MOESM2_ESM.pdf]

nature portfolio

Corresponding author(s): Clifford B. Saper

Last updated by author(s): Mar 3, 2026

Reporting Summary

Nature Portfolio wishes to improve the reproducibility of the work that we publish. This form provides structure for consistency and transparency in reporting. For further information on Nature Portfolio policies, see our Editorial Policies and the Editorial Policy Checklist.

Statistics

For all statistical analyses, confirm that the following items are present in the figure legend, table legend, main text, or Methods section.

n/a	Confirmed	
☐	☒	The exact sample size (*n*) for each experimental group/condition, given as a discrete number and unit of measurement
☐	☒	A statement on whether measurements were taken from distinct samples or whether the same sample was measured repeatedly
☐	☒	The statistical test(s) used AND whether they are one- or two-sided<br>*Only common tests should be described solely by name; describe more complex techniques in the Methods section.*
☒	☐	A description of all covariates tested
☐	☒	A description of any assumptions or corrections, such as tests of normality and adjustment for multiple comparisons
☐	☒	A full description of the statistical parameters including central tendency (e.g. means) or other basic estimates (e.g. regression coefficient) AND variation (e.g. standard deviation) or associated estimates of uncertainty (e.g. confidence intervals)
☐	☒	For null hypothesis testing, the test statistic (e.g. *F*, *t*, *r*) with confidence intervals, effect sizes, degrees of freedom and *P* value noted<br>*Give P values as exact values whenever suitable.*
☒	☐	For Bayesian analysis, information on the choice of priors and Markov chain Monte Carlo settings
☒	☐	For hierarchical and complex designs, identification of the appropriate level for tests and full reporting of outcomes
☒	☐	Estimates of effect sizes (e.g. Cohen's *d*, Pearson's *r*), indicating how they were calculated

Our web collection on statistics for biologists contains articles on many of the points above.

Software and code

Policy information about availability of computer code

Data collection	CRACM recording data were collected using Clampfit 10 (Molecular Devices). Tb and LMA were collected using using ClockLab ActiMetrics software version 6.1.02. Cort levels were read using the plate reader iMark, Bio-Rad, US. Images were acquired using Olympus VS120 slide-scanning microscope and confocal microscope (Leica Stellaris 5).
Data analysis	CRACM recording data were analyzed using Clampfit 10 (Molecular Devices), MiniAnalysis 6 software (Synaptosoft), customized Python scripts (Python 3, www.python.org) and MatLab (version R2020B; MathWorks; Natick, MA) software, Igor Pro version 6 (WaveMetrics) and Prism 7 (GraphPad, La Jolla, CA). Tb, LMA and Cort levels were analyzed using using ClockLab ActiMetrics software version 6.1.02, and GraphPad Prism software version 8. Images were analyzed using Olympus OlyVIA software (3.4.1) or Leica application suite (version 4.2.1).

For manuscripts utilizing custom algorithms or software that are central to the research but not yet described in published literature, software must be made available to editors and reviewers. We strongly encourage code deposition in a community repository (e.g. GitHub). See the Nature Portfolio guidelines for submitting code & software for further information.

Data

Policy information about availability of data

All manuscripts must include a data availability statement. This statement should provide the following information, where applicable:

- Accession codes, unique identifiers, or web links for publicly available datasets
- A description of any restrictions on data availability
- For clinical datasets or third party data, please ensure that the statement adheres to our policy

The data generated in this study are provided in the Source Data file, are stored on Beth Israel Deaconess Medical Center institutional servers and will be made available from the corresponding authors, upon reasonable request.

Research involving human participants, their data, or biological material

Policy information about studies with human participants or human data. See also policy information about sex, gender (identity/presentation), and sexual orientation and race, ethnicity and racism.

Reporting on sex and gender	N/A
Reporting on race, ethnicity, or other socially relevant groupings	N/A
Population characteristics	N/A
Recruitment	N/A
Ethics oversight	N/A

Note that full information on the approval of the study protocol must also be provided in the manuscript.

Field-specific reporting

Please select the one below that is the best fit for your research. If you are not sure, read the appropriate sections before making your selection.

☒ Life sciences ☐ Behavioural & social sciences ☐ Ecological, evolutionary & environmental sciences

For a reference copy of the document with all sections, see nature.com/documents/nr-reporting-summary-flat.pdf

Life sciences study design

All studies must disclose on these points even when the disclosure is negative.

Sample size	Power analysis suggested minimum sample sizes of 6-8 per group. The sample size complied with the ethical guidelines for minimizing animal use, but maintaining reliable measures for the experimental parameter.
Data exclusions	Data were excluded when viral injection missed the target or did not cover at least 70% after histological verification. Outlier Cort measurements higher than 100 ng/ml (under conditions of circadian measurements, without stress or stimulation of CRH neurons) were excluded since they were consider as indicating a stressed condition for the mouse. Exclusion criterion were pre-established.
Replication	Data were collected across multiple animals in at least in two separate cohorts in the majority of the cases, in order to ensure that findings were not biased toward a particular animal or experimental cohort. Every replication attempt was successful.
Randomization	Subjects were allocated to experimental groups randomly.
Blinding	Investigators who excluded animals (e.g., for injection placement) were blinded to the group of the animal. Invetigators were not blinded during group allocation or analysis due to the nature of the experiments. Comparisons were made between conditions within the same animal or with littermate controls. No prior assumptions were made prior to data collection.

Reporting for specific materials, systems and methods

We require information from authors about some types of materials, experimental systems and methods used in many studies. Here, indicate whether each material, system or method listed is relevant to your study. If you are not sure if a list item applies to your research, read the appropriate section before selecting a response.

Materials & experimental systems

n/a	Involved in the study
☐	☒ Antibodies
☒	☐ Eukaryotic cell lines
☒	☐ Palaeontology and archaeology
☐	☒ Animals and other organisms
☒	☐ Clinical data
☒	☐ Dual use research of concern
☒	☐ Plants

Methods

n/a	Involved in the study
☒	☐ ChIP-seq
☒	☐ Flow cytometry
☒	☐ MRI-based neuroimaging

Antibodies

Antibodies used	Chicken anti-GFP (Invitrogen; 1:5,000, A10262), Rat anti-mCherry (Invitrogen; 1:4,000, M11217), Mouse anti-NueN (Millipore; 1:3,000, MAB377), Alexa fluor 488-conjugated Donkey anti-Chicken (Jackson; 1:200, AB2340375), Alexa fluor 555-conjugated Goat anti-Rat (Invitrogen; 1:200, A21434), Alexa fluor 488-conjugated Goat anti-Mouse (Jackson; 1:200, AB2338840).
Validation	The antibodies were previously verified and used to amplify the native fluorescent signal from the fluorescent proteins during the histological verification of injections in this and previous studies of the lab. No signal was seen for GFP or mCherry in uninjected mice. The NeuN antibody was used only as a histological marker for neuronal ablation, and was absent at the injected sites only in animals with ablations.

Animals and other research organisms

Policy information about studies involving animals; ARRIVE guidelines recommended for reporting animal research, and Sex and Gender in Research

Laboratory animals	Vgat-ires-Cre (JAX: 016962), Vglut2-ires-Cre (JAX: 016963), CRH-ires-Cre (JAX: 012704), CRH-VenusΔNeo 43, Vglut2loxP/loxP (JAX:036439), VgatloxP/loxP 44 (JAX: 012897) and R26-loxSTOPlox-L10-GFP mice (Krashes, M. J. et al. 2014).
Wild animals	No wild animal were used
Reporting on sex	Because adult female mice lose their circadian rhythm of Tb during estrus and we needed to record circadian rhythms across many days, and basal Cort levels also changed according to the estrus phase, we used in this study only male adult mice.
Field-collected samples	N/A
Ethics oversight	All procedures were performed in accordance with the National Institutes of Health Guide for the Care and Use of Laboratory Animals, and formal approval of our protocols was obtained from the Institutionary Animal Care and Use Committee at Beth Israel Deaconess Medical Center.

Note that full information on the approval of the study protocol must also be provided in the manuscript.

Plants

Seed stocks	N/A
Novel plant genotypes	N/A
Authentication	N/A
